# A multiepitope vaccine candidate against infectious bursal disease virus using immunoinformatics-based reverse vaccinology approach

**DOI:** 10.3389/fvets.2022.1116400

**Published:** 2023-01-13

**Authors:** Irfan Gul, Amreena Hassan, Jan Mohd Muneeb, Towseef Akram, Ehtishamul Haq, Riaz Ahmad Shah, Nazir Ahmad Ganai, Syed Mudasir Ahmad, Naveed Anjum Chikan, Nadeem Shabir

**Affiliations:** ^1^Laboratory of Vaccine Biotechnology, Division of Animal Biotechnology, Faculty of Veterinary Sciences and Animal Husbandry, Sher-e-Kashmir University of Agricultural Sciences and Technology of Kashmir, Srinagar, India; ^2^Department of Biotechnology, University of Kashmir, Srinagar, India; ^3^Division of Computational Biology, Daskdan Innovations Pvt. Ltd., Srinagar, India

**Keywords:** IBDV, immunoinformatics, B cell epitopes, T cell epitopes, molecular dynamics simulations

## Abstract

Infectious bursal disease virus is the causative agent of infectious bursal disease (Gumboro disease), a highly contagious immunosuppressive disease of chicken with a substantial economic impact on small- and large-scale poultry industries worldwide. Currently, live attenuated vaccines are widely used to control the disease in chickens despite their issues with safety (immunosuppression and bursal atrophy) and efficiency (breaking through the maternally-derived antibody titer). To overcome the drawbacks, the current study has, for the first time, attempted to construct a computational model of a multiepitope based vaccine candidate against infectious bursal disease virus, which has the potential to overcome the safety and protection issues found in the existing live-attenuated vaccines. The current study used a reverse vaccinology based immunoinformatics approach to construct the vaccine candidate using major and minor capsid proteins of the virus, VP2 and VP3, respectively. The vaccine construct was composed of four CD8^+^ epitopes, seven CD4^+^ T-cell epitopes, 11 B-cell epitopes and a Cholera Toxin B adjuvant, connected using appropriate flexible peptide linkers. The vaccine construct was evaluated as antigenic with VaxiJen Score of 0.6781, immunogenic with IEDB score of 2.89887 and non-allergenic. The 55.64 kDa construct was further evaluated for its physicochemical characteristics, which revealed that it was stable with an instability index of 16.24, basic with theoretical pI of 9.24, thermostable with aliphatic index of 86.72 and hydrophilic with GRAVY score of −0.256. The docking and molecular dynamics simulation studies of the vaccine construct with Toll-like receptor-3 revealed fair structural interaction (binding affinity of −295.94 kcal/mol) and complex stability. Further, the predicted induction of antibodies and cytokines by the vaccine construct indicated the possible elicitation of the host's immune response against the virus. The work is a significant attempt to develop next-generation vaccines against the infectious bursal disease virus though further experimental studies are required to assess the efficacy and protectivity of the proposed vaccine candidate *in vivo*.

## Graphical Abstract

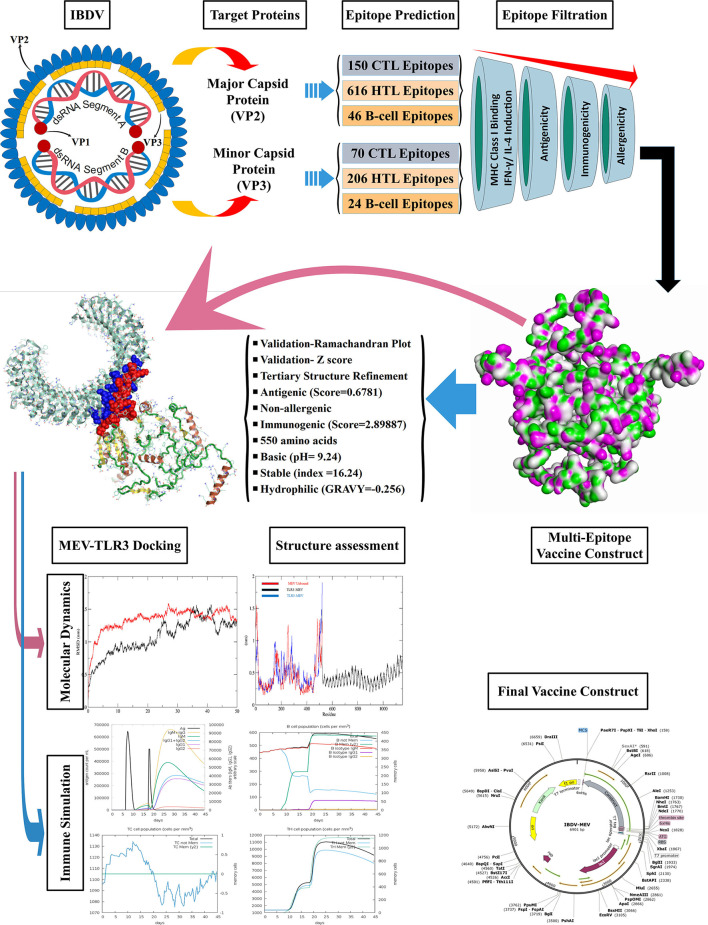

## Introduction

Infectious bursal disease (IBD) is an economically significant and contagious poultry disease. IBD, also known as Gumboro disease, is caused by the double-stranded RNA virus (dsRNA) known as infectious bursal disease virus (IBDV). The virus belongs to the family Birnaviridae, replicates in the bursa of Fabricius (BF) in young chickens causing depletion of B-lymphocytes (1, 2). As a result, young chickens with IBD have significant immunosuppression, putting them at risk to secondary infections (3). IBDV is a non-enveloped virus with icosahedral symmetry and a bi-segmented dsRNA genome (4, 5). Segment A of the genome contains two partially overlapping larger and smaller open reading frames (ORFs). The larger ORF produces a 110 kDa polyprotein that self-processes into two structural capsid proteins (VP2 and VP3) and a non-structural protease protein (VP4), with molecular weights of 48, 33–35, and 24 kDa, respectively (6, 7). The smaller ORF encodes VP5 polypeptide (8), a non-structural protein not required for viral replication *in vitro* but crucial for virus release. The VP2 polypeptide forms the major capsid of IBDV and carries the main immune determinants for eliciting neutralizing antibodies (9). Due to the considerable conservation of the VP2 amino acid sequence across IBDV strains, the linear epitopes have been identified at the residue level. However, the conformation-dependent epitopes are characterized by the core area covering amino acid residues 206–350, the only place where antigenic alterations have been found. The minor capsid protein VP3 is a group-specific immunogenic antigen, with the earliest antibodies appearing after IBDV infection directed at VP3 (10). Segment B of the viral genome encodes for the non-structural protein VP1 (97 kDa), the RNA-dependent RNA polymerase (RdRp) (11). Bound to the genomic RNA, the RdRp stays enclosed within the viral particle.

Adequate control of IBD is possible only by following vaccination regimes as the highly contagious IBDV is a very resilient and persistent virus that survives in poultry houses despite stringent disinfection (12). Despite the many advantages present-day IBD vaccinations (Live attenuated vaccines; LAVs) provide, further improvement is warranted for various reasons. The efficacy of LAVs has been found to decrease in the presence of maternally derived antibodies (MAb) which protect the young chicken during the first few weeks (13, 14). Besides poor efficacy in the presence of MAb, they also possess serious safety issues as they cause varying degrees of bursal atrophy and degeneration as well, in addition to the emergence of antigenic variants in vaccinated flocks, particularly very virulent strains (15–17).

Multiepitope-based vaccines (MEV) are peptide-based vaccines that consist of T cell and B cell epitopes and have the ability to trigger efficient cellular and humoral immune responses (18). MEV can prove a promising strategy for combating viral infections, potentially eliciting a broad immune response due to T cell receptor (TCR) recognized Major Histocompatibility Complex (MHC)-restricted epitopes from target antigens. Moreover, MEV offers improved immunogenicity and long-lasting immune responses without any immunization-related side effects compared to traditional vaccines (19–25). Although the MEV with such advantages have the potential to prove powerful prophylactic and therapeutic agents, the screening of appropriate target antigens and their immunodominant epitopes, as well as the development of an effective delivery system, continue to be the current challenges of MEV design. Therefore, the development of an effective MEV depends on selecting suitable candidate antigens and the immunodominant epitopes associated with them (26–28). Hence this study aimed to develop a potential MEV against IBDV by targeting major and minor capsid proteins through immunoinformatics, molecular modeling and reverse vaccinology approaches.

## Materials and methods

### The retrieval of protein sequences

The VP2 and VP3 protein sequences from 10 distinct IBDV strains (Supplementary Table 1) were obtained in FASTA format from the National Center for Biotechnology Information (NCBI) protein database (https://www.ncbi.nlm.nih.gov/protein). Multiple sequence alignment was performed on the reference sequences obtained from NCBI using DNA star (DNASTAR, Inc.Madison, WI, USA) with ClustalW parameters. The antigenicity of the reference sequences was evaluated using the VaxiJen v2.0 Server, using a 0.4 antigenicity threshold (http://www.ddg-pharmfac.net/VaxiJen/VaxiJen/VaxiJen.html) (29).

### T-cell epitopes Identification

In this study, human HLA alleles were considered instead of chicken HLA alleles because of the unavailability of the applicable data. Consequently, human-related data was utilized to predict the MHC epitopes of selected sequences (30, 31). Humans and chickens have distinct MHC alleles; however, it has been reported that MHC haplotype anchor residue regions in both species are comparable (32).

### Cytotoxic T-cell (CTL/CD8^+^) epitope prediction

The sequences which were found to be antigenic were further submitted to the NetCTL v1.2 server (https://services.healthtech.dtu.dk/service.php?NetCTL-1.2) for the generation of nine amino acid long fragments (33). The fragments were filtered based on interactions with the MHC class I HLA alleles and the production of the CD8^+^ T cell response. The Stabilized Matrix Base Method (SMM) prediction method of the IEDB tool (http://tools.iedb.org/mhci/) was used to identify the MHC-I HLA binding CTL/CD8^+^ epitopes out of the resulting nine amino acid long fragments (34). The parameters were set to human as the MHC source species, amino acid length of 9, and IC50 value <250. The screened epitopes were evaluated for antigenicity using the VaxiJen v2.0 server with a 0.5 antigenicity threshold. The potential antigens were further subjected to the IEDB MHC-I immunogenicity tool (http://tools.iedb.org/immunogenicity/) for the evaluation of immunogenicity (35).

### Helper T-cell (HTL/CD4^+^) epitope prediction

The antigenic consensus VP2 and VP3 sequences were submitted to the IEDB MHC-II binding tool (http://tools.iedb.org/mhcii/) to predict HTL epitopes interacting with MHC class II HLA alleles (36). The allele length was adjusted to 15 and the IC50 threshold to 250 to filter out probable epitopes. The screened epitopes were subsequently evaluated for potential IFN-γ cytokine induction using the IFN epitope tool (http://crdd.osdd.net/raghava/ifnepitope/) with SVM (support vector machine) approach and the IFN vs. non-IFN predictive models (37). Moreover, the IL4 inducer epitopes were identified using the IL4pred tool (http://crdd.osdd.net/raghava/il4pred/) (38). The selected epitopes were then assessed for antigenicity using immunoinformatic techniques identical to those used to test CTL epitopes.

### Linear B-cell epitope prediction

The antigenic consensus sequences were subjected to the ABCPRED server (https://webs.iiitd.edu.in/raghava/abcpred/) to identify antigens that can trigger the production of antibodies by eliciting a B cell immune response. The server predicts linear B cell epitopes using an artificial neural network (39). The potential epitopes were screened based on the prediction parameters selected: the window length of 16 and the threshold value of 0.51.

### Conservancy and allergenicity assessment

The selected T cell and B cell epitopes determined to be immunogenic and antigenic were evaluated for conservancy using the IEDB conservation across antigen tool (http://tools.iedb.org/conservancy/) (40). The AllerTop v2.0 tool (https://www.ddg-pharmfac.net/AllerTOP/) was used to assess the allergenicity of the conserved epitopes and identify the non-allergic epitopes (41, 42).

### Vaccine design and assessment

The top candidates for CD8^+^, CD4^+^, and B cell epitopes were identified using several immunoinformatic tools, as indicated above. To construct the IBD-MEV, these epitopes coupled with an adjuvant were linked with appropriate linker peptides. The CD8^+^/CTL epitopes were linked using an AAY linker, and the CD4^+^/HTL were connected using GPGPG linkers. The HEYGAEALERAG linker was used to join CTL epitopes with HTL epitopes, while the B cell epitopes were linked by KK linkers. An appropriate adjuvant, cholera toxin subunit B (CTB), was incorporated to the N terminal of the construct peptide using the EAAK linker. The adjuvant was included to enhance the immunogenicity of the vaccine construct (43). The MEV construct was assessed for antigenicity and allergenicity using the VaxiJen v2.0 server and AllerTop v2.0 server, respectively. At the same time, the ProtParam53 web server (https://web.expasy.org/protparam/) determined the physical and chemical characteristics, such as the molecular weight (kDa), the number of amino acid residues, the theoretical isoelectric point (pI), the estimated half-life, the instability index, the aliphatic index, the hydropathicity, and grand average of hydropathicity (GRAVY) (44).

### Secondary and Tertiary structure prediction and validation

The primary sequence of the final construct was subjected to the PSIPRED web tool (http://bioinf.cs.ucl.ac.uk/psipred/) for prediction and analysis of the secondary structure (45). While the AlphaFold2-based Colabfold was employed to predict and generate the tertiary structure of the vaccine construct (46, 47). To enhance the quality of the structure, the predicted tertiary structure was subjected to molecular refinement with the aid of the GalaxyRefine server (http://galaxy.seoklab.org/cgi-bin/submit.cgi?type=REFINE) (48). The resulting models were screened using the GDT-HA, RMSD, and MolProbity scores to choose the most refined model, which was then verified using the Ramachandran plot and ProSA-web-predicted Z-score (https://prosa.services.came.sbg.ac.at/prosa.php) (49, 50).

### Docking and molecular dynamic simulation analysis

The vaccine construct was docked with the Toll Like Receptor 3 (TLR3; PDB ID: 1ZIW) using the HDOCK server (http://hdock.phys.hust.edu.cn/) with default parameters (51). The server provides 10 poses for each docking run, wherein the model with the lowest binding energy was selected and visualized by PyMOL (https://pymol.org/) and Discovery Studio Biovia 2021 (https://discover.3ds.com/). Molecular Dynamics Simulation by GROMACS 2021.1 was performed using OPLS-AA/L all-atom force field to study the stability of the complex (52). The complex was placed in a unit cell, defined as a 1-nm cube, solvated with water using a solvate model. Ions were added according to the charge present on the vaccine construct, and the obtained electro-neutral structure was relaxed through energy minimization. The equilibrating of the water around the complex was conducted under NVT and NPT conditions for 100 ps. The temperature was set to a maximum of 300 K. Following the equilibration phases, MD simulation data was collected to perform the 50 ns final run with a time step of 2 fs at constant pressure (1 bar) and temperature (300 K). The resulting trajectories were analyzed using the inbuilt utilities of GROMACS.

### *In silico* cloning and optimization of vaccine construct

Using the Java Codon Adaptation Tool (http://www.jcat.de/), the vaccine construct was codon optimized using the *Escherichia coli* K12 strain as the host organism. The JCat adaptation was defined using the codon adaptation index (CAI) and GC content of the optimizes sequence (53). The ideal CAI score of an edited gene sequence is around 0.8 and 1.0, with a GC percentage of 30%−70%, suggesting better gene expression in the associated organism with no translation mistakes (54). To the optimized vaccine sequence, restriction sites *BamHI* (GGATCC) was added to the 5′ end while *XhoI* (CTCGAG) restriction site was added to the 3′ end. SnapGene Viewer V3.2.1 (http://www.snapgene.com/) carried out the *in-silico* cloning of the optimized vaccine sequence into the pET-28a (+) expression vector system.

### *In silico* immune simulation of vaccine construct

The C-ImmSim server (https://kraken.iac.rm.cnr.it/C-IMMSIM/) was used to model and evaluate the immune response of the vaccine construct for the specified vaccination program (55). A vaccination without lipopolysaccharide (LPS) was used for the simulation, and all other parameters were left at their default values. A single injection of the vaccine construct was administered at two intervals; Day 7 and Day 18.

## Results

### Cytotoxic T-cell (CTL/CD8^+^) epitope prediction

The VP2 and VP3 consensus sequences were subjected to the NetCTL v1.2 server to predict specific immunogenic CTL epitopes. A total of 150 and 70 nonamer epitopes were obtained from VP2 and VP3 proteins, and each of them had a considerable binding affinity for the 12 superfamily HLA alleles. Using the IEDB MHC-I prediction tool, the CTL epitope nonamers were scrutinized for specific MHC-I binding affinity with the SSM-based method. The epitopes were filtered by the IC50 value parameter (<250), yielding 86 VP2 and 37 VP3 CTL epitopes. The screened epitopes were examined with the VaxiJen v2.0 server for antigenicity (threshold ≥0.5). Among the predicted epitopes of VP2 and VP3 proteins, 47 and 16 epitopes showed considerable antigenic potential, with the highest antigenic score of 1.6076 and 0.8179 for VP2 epitope “TSYDLGYVR” and VP3 epitope “EAAANVDPL.” Following the assessment of immunogenicity using the IEDB tool, the epitopes were filtered to 26 VP2 and 8 VP3 immunogenic epitopes. Finally, the allergenicity and conservancy analysis was carried out using the AllerTop v2.0 server and IEDB conservation across antigen tool, where the allergenic and non-conserved epitopes were screened out, and only 15 VP2 and 2 VP3 CTL epitopes were regarded as concluding predicted epitopes (Table 1).

**Table 1:** Final predicted cytotoxic T cells (CD8^+^/CTL) epitopes.

	**Epitopes**	**Position**	**HLA allele**	**ic50**	**Immunogenicity**	**Antigenicity**	**Allergenicity**
VP2	KTVWPTREY	18	HLA-A*30:02	117.8853942	0.36421	0.6857	Non-allergen
			HLA-B*15:02	123.6602158			
	LKIAGAFGF	124	HLA-B*15:02	191.9685126	0.271	0.9619	Non-allergen
	VLVGEGVTV	29	HLA-A*02:01	197.2922383	0.23442	0.5956	Non-allergen
			HLA-A*02:06	66.76517628			
	GIKTVWPTR	47	HLA-A*31:01	51.8167297	0.23109	0.8973	Non-allergen
	YGRFDPGAM	90	HLA-B*15:02	114.3483757	0.19722	1.1414	Non-allergen
			HLA-B*35:01	147.4280088			
	RLGDPIPAI	23	HLA-A*02:01	113.7915466	0.1613	0.7382	Non-allergen
			HLA-A*02:06	194.3345415			
	SYDLGYVRL	54	HLA-B*15:02	47.44932078	0.09064	1.5198	Non-allergen
	TSYDLGYVR	49	HLA-A*31:01	16.27308901	0.06322	1.6076	Non-allergen
			HLA-A*68:01	16.92349276			
			HLA-A*11:01	87.96299338			
	GEGVTVLSL	108	HLA-B*40:02	133.0025599	0.02318	0.5789	Non-allergen
			HLA-B*15:02	179.982384			
			HLA-B*40:01	58.1969199			
VP3	KVYEVNHGR	16	HLA-A*68:01	54.88959167	0.18076	0.773	Non-allergen
			HLA-A*11:01	110.9941271			
			HLA-A*31:01	7.878980035			
	EAAANVDPL	29	HLA-A*68:02	35.52957397	0.09687	0.8179	Non-allergen
			HLA-B*15:02	54.47910949			
			HLA-B*35:01	125.1929866			

The epitopes were predicted using NetCTL v1.2 and scrutinized using the IEDB MHC-I prediction tool.

### Helper T-cell (HTL/CD4^+^) epitope prediction

Overall, 616 VP2 and 206 VP3 15-mer HTL epitopes were identified using IEDB MHC-II binding tool screening out *via* filtration based on IEDB tool IC50 value (< 250) and VaxiJen tool antigenicity score (≥0.4). The 15-mer epitopes were further examined for IFN-gamma and interleukin inducer properties using IFN epitope and IL-4pred immunoinformatic tools. A total of 99 VP2 and 100 VP3 CD4^+^ T cell epitopes exhibited the property to induce IFN-γ, while only 25 VP2 epitopes and 16 VP3 epitopes exhibited IL-4 inducer properties. Finally, the antigenicity and allergenicity analysis was carried out using VaxiJen and AllerTop v2.0 servers, where the non-antigen and allergenic epitopes were screened out. 6 VP2 epitopes and 1 VP3 epitope were concluded as the most promising HTL epitope candidates for the final vaccine construct (Table 2).

**Table 2:** Final predicted Helper T cells (CD4^+^/HTL) epitopes.

	**Epitopes**	**HLA allele**	**ic50**	**Immunogenicity**	**Antigenicity**	**Allergenicity**
VP2	SEITQPITSIKLEIV	HLA-DRB1*07:01	112	0.03638	0.5556	Non-allergen
		HLA-DRB1*01:01	183			
	LGYVRLGDPIPAIGL	HLA-DRB1*01:01	151	0.41149	1.1488	Non-allergen
	DLGYVRLGDPIPAIG	HLA-DRB1*01:01	170	0.36082	1.3044	Non-allergen
	YDLGYVRLGDPIPAI	HLA-DRB1*01:01	173	0.28928	1.3085	Non-allergen
	TSYDLGYVRLGDPIP	HLA-DRB1*01:01	181	0.2418	1.4101	Non-allergen
	SYDLGYVRLGDPIPA	HLA-DRB1*01:01	186	0.25524	1.3072	Non-allergen
VP3	ELESAVRAMEAAANV	HLA-DRB1*04:04	37	0.0635	0.4095	Non-allergen
		HLA-DRB1*04:01	172			
		HLA-DRB1*01:01	37			

The epitopes were predicted using IEDB MHC-II prediction tool and screened as IFN-gamma and interleukin inducers.

### Linear B-cell epitope prediction

An iitd.edu.in server was used to generate 46 VP2 and 24 VP3 B cell epitopes. Out of these, 28 VP2 and 10 VP3 epitopes were revealed as antigenic by the VaxiJen server (threshold >0.5). The Immunogenicity analysis further filtered the epitopes to 14 VP2 and 4 VP3 Immunogenic epitopes. Among the immunogenic epitopes, only 9 VP2 and 3 VP3 B cell epitopes were assessed as non-allergenic and selected for the final vaccine construct, omitting the allergenic epitopes (Table 3).

**Table 3:** Final selected linear B-cell epitopes.

	**Epitopes**	**Position**	**Immunogenicity**	**Antigenicity**	**Allergenicity**
VP2	DRLGIKTVWPTREYTD	398	0.43501	0.736	Non-allergen
	GYVRLGDPIPAIGLDP	168	0.42048	0.8952	Non-allergen
	NLTVGDTGSGLIVFFP	38	0.3763	0.8846	Non-allergen
	GSVVTVAGVSNFELIP	352	0.35349	0.9296	Non-allergen
	KNLVTEYGRFDPGAMN	373	0.34016	0.5987	Non-allergen
	LILSERDRLGIKTVWP	392	0.19243	1.0791	Non-allergen
	GLTAGTDNLMPFNIVI	273	0.18982	0.8909	Non-allergen
	NSPLKIAGAFGFKDII	425	0.16852	0.7304	Non-allergen
	TSEITQPITSIKLEIV	290	0.15834	0.5207	Non-allergen
VP3	GVEARGPTPEGAQREK	100	0.33321	0.5198	Non-allergen
	TPEWVALNGHRGPSPG	132	0.30795	0.7318	non-allergen
	PTPEGAQREKDTRISK	106	0.11713	0.5853	Non-allergen

The epitopes were predicted using the abcpred web tool.

### Vaccine design and assessment

**Figure 1:**
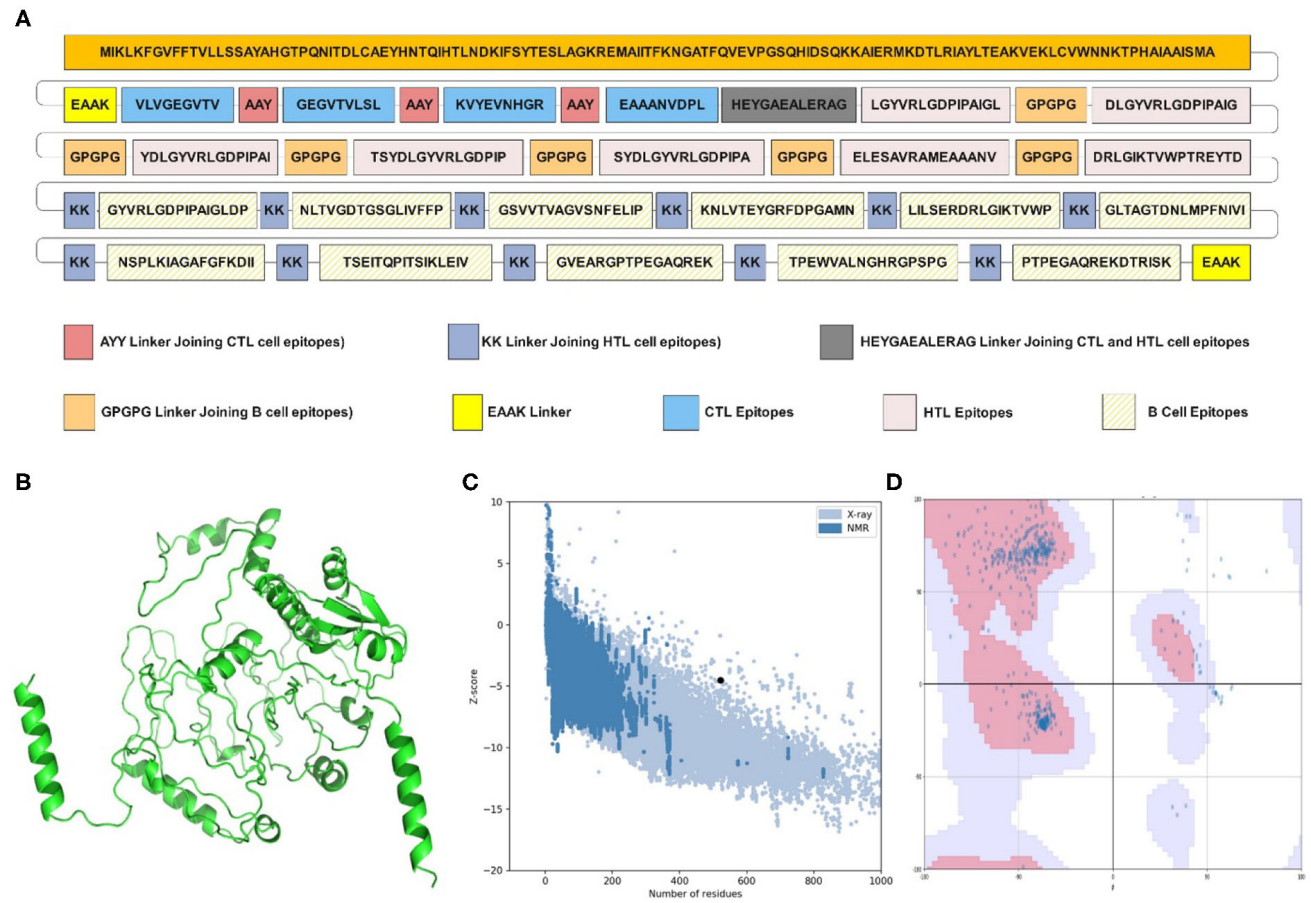
Structural analysis and validation of designed vaccine. **(A)** Schematic design of the final vaccine construct. AAY Linkers join the CTL epitopes, HTL epitopes are joined by GPGPG linkers and B-cell epitopes by KK linkers. The Cholera Toxin B (CTB) adjuvant is added to the N-terminus of the sequence by an EAAK linker. An additional EAAK linker C-Terminal and HEYGAEALERAG linkers between CTL and HTL epitopes were incorporated. **(B)** The refined three-dimensional structure of vaccine construct; **(C)** ProSA-web assessment of the vaccine tertiary structure. The evaluation revealed a *Z*-score of −4.49, indicating good quality. **(D)** Ramachandran plot analysis of the refined structure. The evaluation revealed that 96.0% of the residues of the vaccine are present in the favored region.

The epitopes were combined to construct the MEV candidate against IBDV based on their antigenicity, immunogenicity, non-allergenic and non-overlapping characteristics. The final IBD-MEV design included 4 CTL, 7 HTL, 11 linear B cell epitopes, and a CTB adjuvant, with AAY linkers connecting the CTL, GPGPG linkers connecting the HTL, and KK linkers connecting the B cell epitopes. The CTB adjuvant was attached in the N-terminal by an EAAK linker to increase the immunogenicity of IBD-MEV. Moreover, the HEYGAEALERAG linker was inserted between CTL and HTL epitopes, and an EAAK liner was added to the C-terminal of the IBD-MEV construct (Figure 1A). The 522-residue IBD-MEV construct with a molecular weight of 55.64 kDa was evaluated for antigenicity, immunogenicity, and allergenicity, in addition to physical and chemical properties. The vaccine was demonstrated as antigenic (VaxiJen score = 0.6781), immunogenic (score = 2.89887), and non-allergenic. The assessment of the physicochemical properties using the ProtParam server presented that the IBD-MEV construct has a theoretical isoelectric point (PI) of 9.24, making it substantially basic. The IBD-MEV construct was determined to be stable with an instability index of 16.24, thermostable with an aliphatic index of 86.72, and hydrophilic with GRAVY scores of −0.256.

### Secondary and tertiary structure prediction and validation

The PSIPRED server examined the secondary structural properties of the IBD-MEV. Accordingly, the construct had 22.22% of the amino acids in the α-helix conformation and 23.56% of amino acids in the β-strand conformation and 54.22% in coil structure conformations (Supplementary Figure 1). The tertiary structure of the IBD-MEV construct was predicted using the AlphaFold2-based Colabfold, while the GalaxyRefine server was employed to refine the structure (Figure 1B). The refined model was obtained with a GDT-HA score of 0.8582, an RMSD value of 0.682, a MolProbity score of 1.459 and a rotamer score of 0.7, indicating the high quality of the model. The preferred refined model structure was validated using the Ramachandran plot, with 96.0% residues in the favored region (Figure 1C). The model was further validated using ProSA-web and has a half-life of 30 h in mammalian reticulocytes (*in vitro*), >20 h in yeast, and >10 h in *E. coli* (*in vivo*). Moreover, a *Z*-score of −4.49 was obtained, signifying the high quality of the structure (Figure 1D). The structural assessment of the IBD-MEV tertiary structure is displayed in Supplementary Figures 1B–F).

### Docking and molecular dynamic simulation analysis

**Figure 2:**
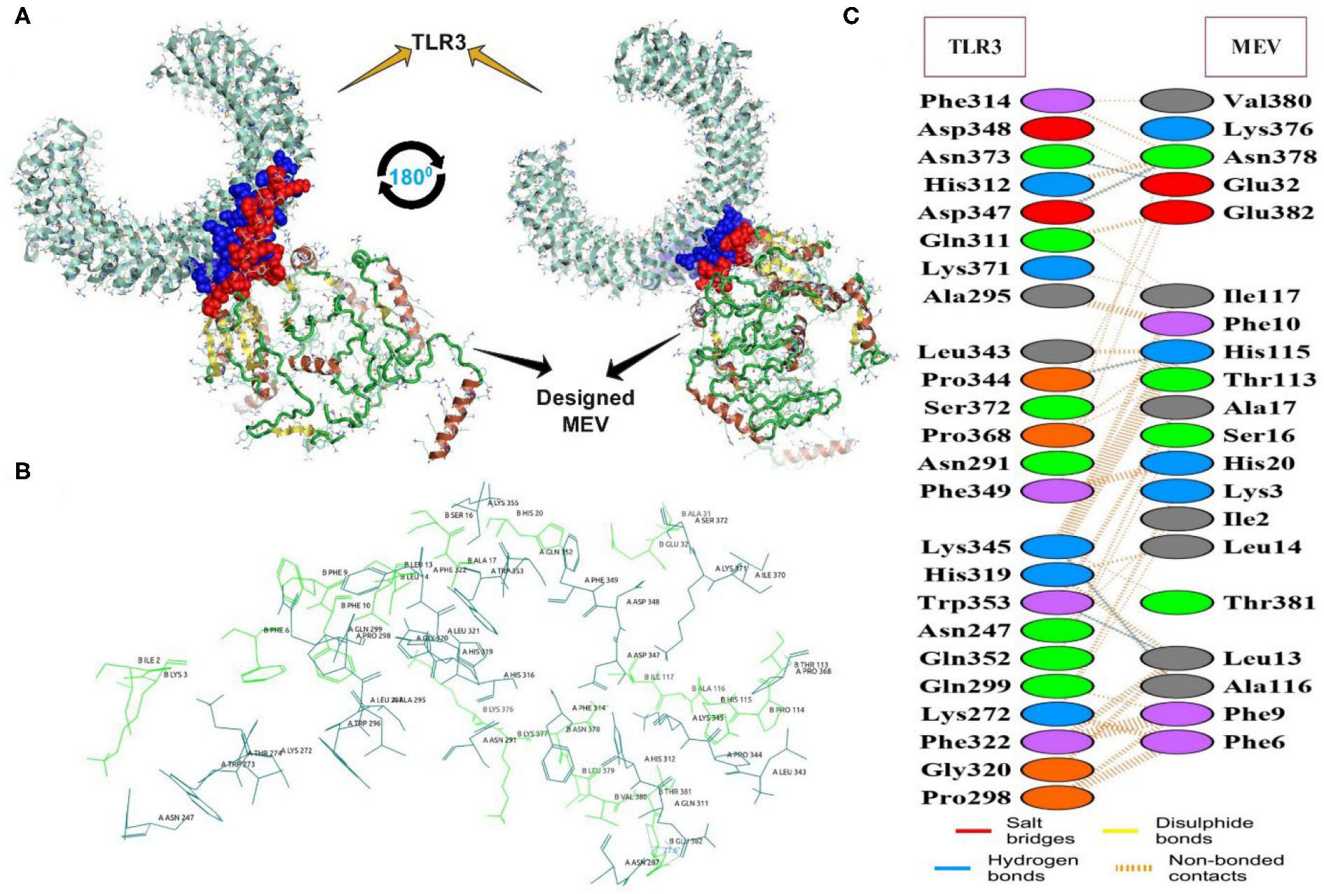
The molecular interaction analysis of the designed MEV with TLR3 after protein-protein docking. **(A)** Interacting tertiary structure whereby interacting residues are shown by blue (TLR3) and red (MEV); **(B)** The interacting residues; TLR3 (Teal) and MEV (Green); **(C)** Different interaction between the interacting residues of TLR3 and MEV.

**Figure 3:**
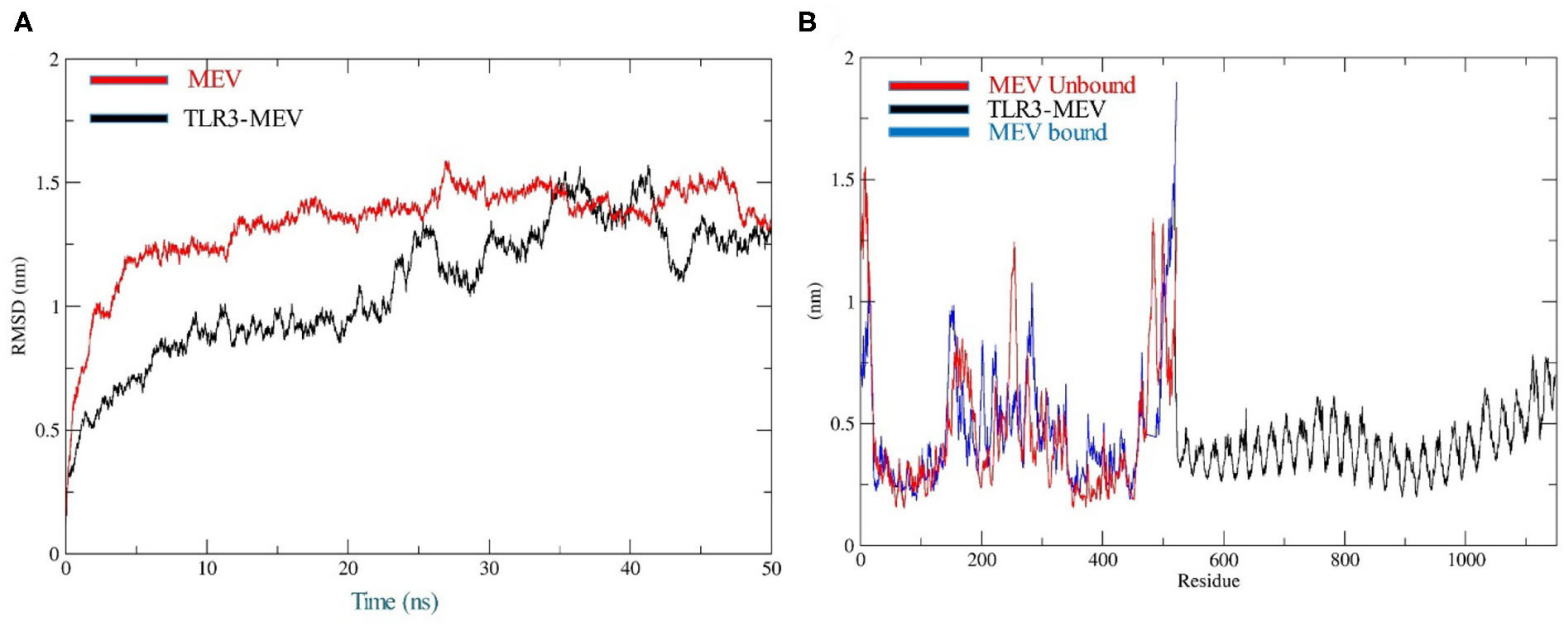
Molecular dynamics simulation analysis at 50-ns MD simulation. **(A)** Analysis of RMSD trajectories for MEV-TLR3 complex (Black) and MEV (Red), relative to the backbone. The RMSD plot showed structural stability of the complex with minimum deviations; **(B)** Analysis of RMSF trajectories for MEV-TLR3 complex (Black), MEV (Red) and MEV bound (Blue) to TLR3. The RMSF plot shows the flexibility of interacting side-chain regions.

The docking of the IBD-MEV construct was performed with TLR3 as a receptor using the HDOCK server. TLR3 is a significant TLR family member recognizing viral double-stranded RNA. The produced docked models were visualized using the PyMOL and Discovery Studio Biovia 2021. The HDOCK returned models were screened based on the binding affinity, and the model with Δ*G* value of −295.94 kcal/mol was selected (Figure 2). The interacting residues of TLR3 and MEV reveal various types of interaction between the two structures (Figure 2C). The complex was subjected to MD simulations to assess the docked complex's stability, binding and dynamics (Supplementary Movie). The backbone RMSD and residue-wise RMSF trajectories were analyzed throughout the 50 ns simulation. The comparison of RMSD fluctuation for backbone atoms of IBD-MEV before docking and MEV-TLR3 complex signifies the stability of the MEV system due to the binding of MEV to the TLR3 (Figure 3A). RMSF analysis (Figure 3B) revealed slight fluctuation in docked complex side-chain atoms, which may reflect high interaction between the IBD-MEV and TLR3.

### *In silico* cloning and optimization of vaccine construct

**Figure 4:**
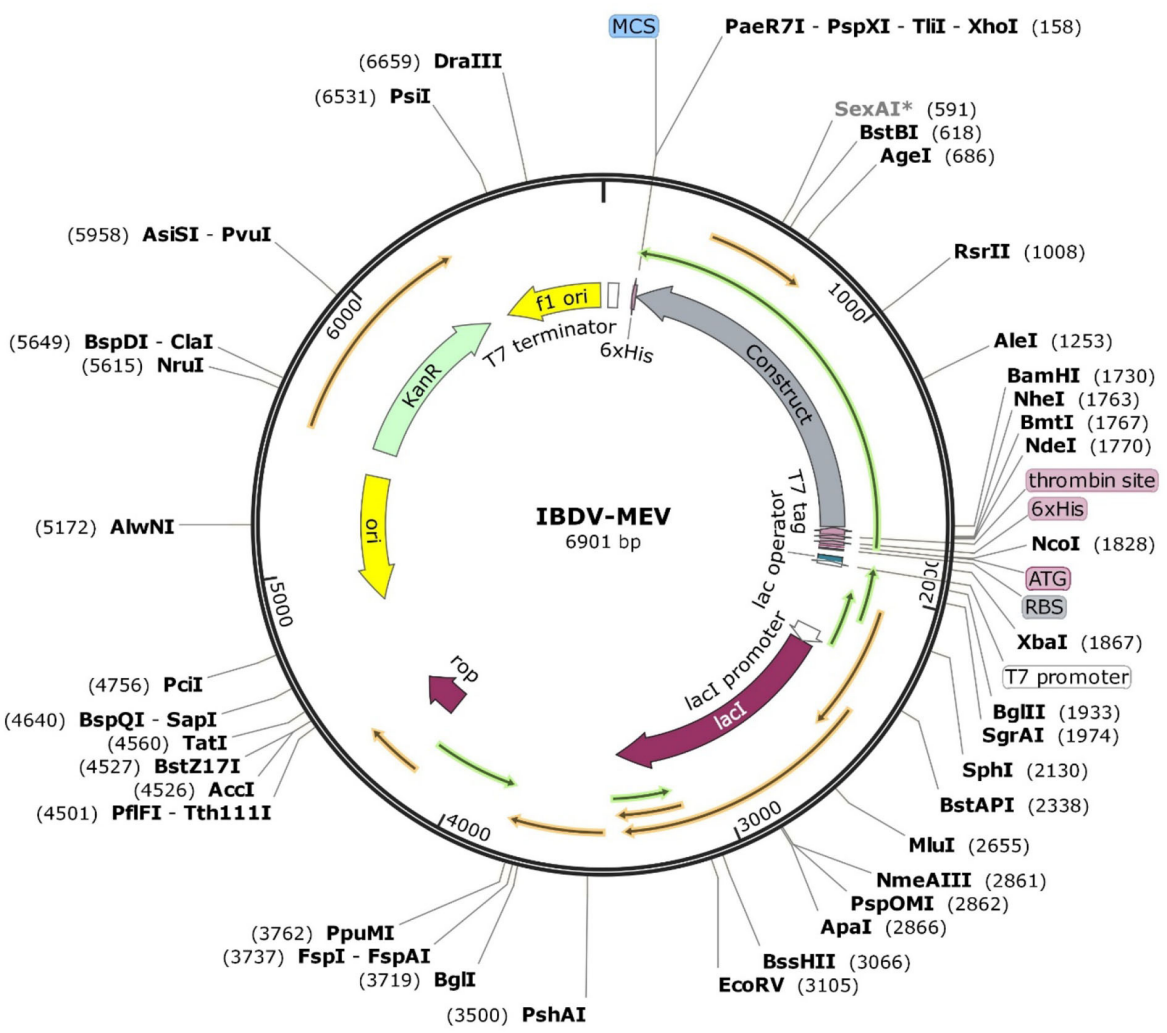
*In silico* restriction cloning of the optimized sequence of the designed MEV into the pET28a(+) expression vector. The MEV construct is labeled as *Construct*, and the restriction sites are incorporated at N-terminal (*XhoI*) and C-terminal (*BamHI*) of the vaccine construct.

The JCat server optimized the codon usage for maximal expression of the IBD-MEV construct according to *E. coli* (strain K12). The obtained CAI value of 0.99 and GC-content of 52.36% imply the effectiveness of IBD-MEV expression in the selected host. The predicted DNA sequence of the IBD-MEV construct was cloned into the pET-28a(+) expression system using the SnapGene. *BamHI* restriction sequence was incorporated at the N-terminal, and *XhoI* site was incorporated at the C-terminal of the construct (Figure 4).

### *In silico* immune simulations of vaccine construct

**Figure 5:**
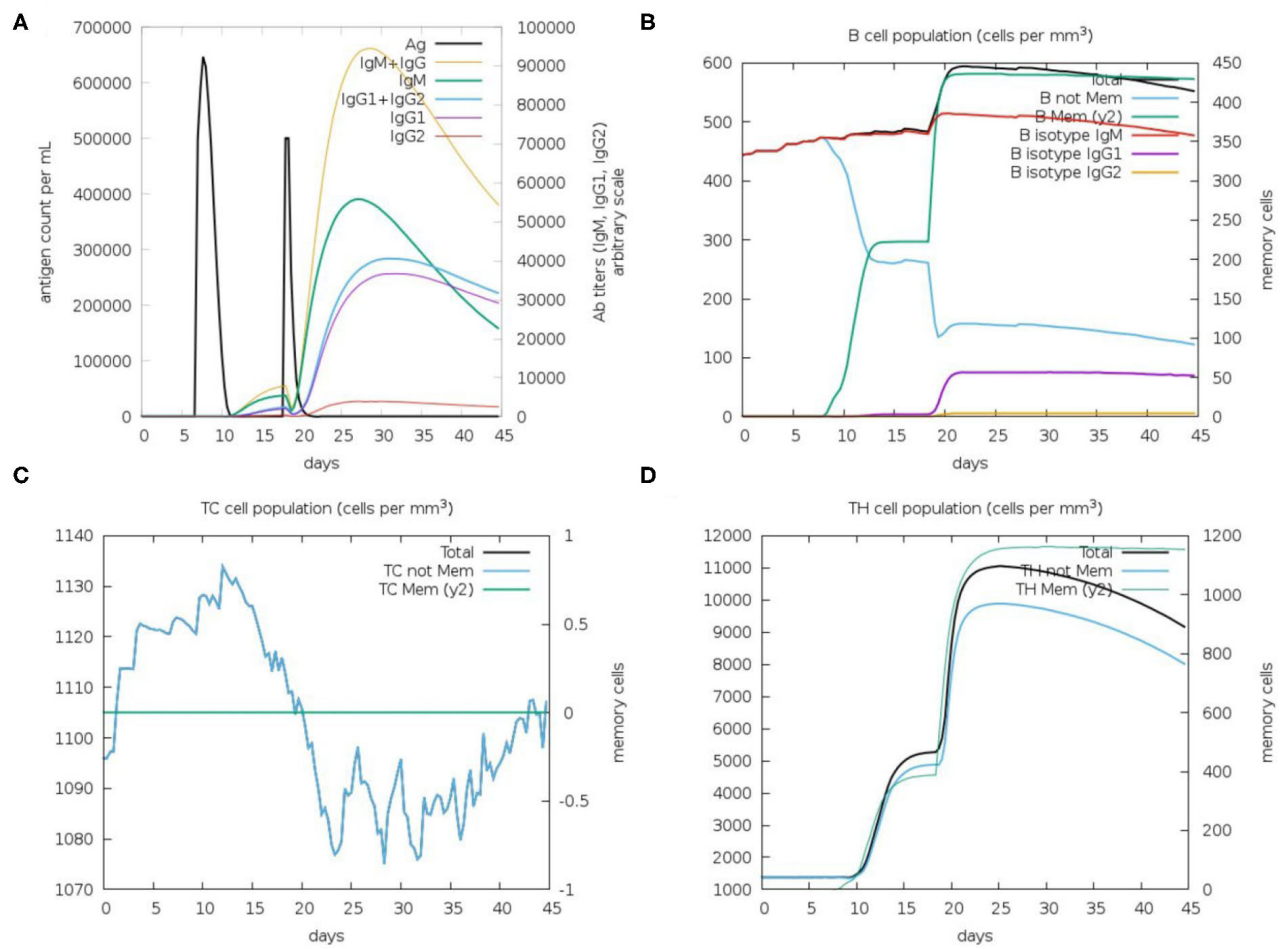
C-ImmSim *in silico* immune simulation analysis, showing immune response against IBDV-MEV construct. **(A)** Immunoglobin production (colored peaks) in response to vaccine injections (black; 7 and 18 Day); **(B)** Amount of B lymphocytes composed of B memory (y2) and B-isotypes (IgM, IgG1, and IgG2) **(C)** CD8^+^ T-cytotoxic lymphocytes (CTL) cell populations and **(D)** CD4^+^ T-helper lymphocytes (HTL) cell population; per state in response to antigen injection.

Immunological simulation findings confirmed various immune profiles created by the vaccination, with the vaccine inducing an immune response *via* an increase in antibodies after delivery to the simulation. The vaccine doses were administered in two intervals: the first dose for a 7-day old chick and the second after 11 days of the first dose (Day 18). The immune response was studied for 45 days. With C-ImmSim simulation, in comparison to the primary reaction indicated by IgM, delivery of the IBD-MEV construct resulted in a considerable increase in the tertiary immune response. After receiving the vaccination, the B cell population produced memory cells that would keep the memory if the host became reinfected (Figure 5). The existence of antibodies that successfully preserved the likelihood of an antigenic rush was confirmed by the drop in antigen level with each vaccination.

## Discussion

IBDV is one of the top infectious issues affecting young chickens, with a significant socio-economic impact on the poultry industry with direct and indirect losses (5). Direct losses include morbidity and mortality losses, while indirect losses owe to immunosuppression-induced secondary infections. Since the virus targets the B cells in the BF, chickens typically display immunosuppression, are less responsive to vaccination campaigns, and are more vulnerable to secondary infections. The use of a live attenuated (mild strain) of IBDV is a frequent IBDV vaccination regimen. LAVs imitate infection to induce host immunity and reduce clinical illness or immunosuppression. Even though this treatment prevents clinical indications of the disease, it produces bursal damage. Moreover, there is a risk that the LAVs may revert to a virulent strain, resulting in bursal injury and immunosuppression (15, 16). LAVs are also ineffective against vvIBDV and rapidly neutralized by MAb (30). Recently, the focus has shifted to developing epitope-based vaccines due to their superior safety profiles and logistical manageability. The potential benefits of epitope-based vaccination are improved safety, time-saving, ability to focus on conserved epitopes and specifically engineer epitope combinations for increased potency (56). As a result, rational selections are made to isolate and separate the ingredients needed for the intended immune response using immuno-informatics approaches to vaccine development. The immunoinformatics techniques can be employed to design proper protein antigens that elicit antibody response and cell-mediated immunity. Therefore, this study aimed to construct a potential MEV against IBDV by focusing on the two capsid proteins of the virus, VP2 and VP3. The trimeric form of VP2 makes up the IBDV virion's major capsid, while the dimeric VP3 subunits make up the inner minor capsid. VP2 is preferably targeted in IBDV vaccine development strategies because it is essential for selection, entry into target cells, and induction of protective, neutralizing antibodies (9). VP3 has also been identified as a putative antigen for the production of a multiepitope vaccine (10). The MEVs would mitigate any potential negative consequences of employing the entire virion, reducing the likelihood of reversion to virulence and other vaccine-related adverse effects.

T cell epitopes are antigenic peptides recognized by the TCR when bound to MHC molecules. MHC class I presents CTL, and MHC class II presents HTL epitopes recognized by CD8^+^ and CD4^+^ T cells, respectively. After identifying the target epitope, CD8^+^ T cells mature into CTL, which can destroy malignant or infected cells. In contrast, CD4^+^ T cells mature into HTL, stimulating B cells to generate antibodies and macrophages to eliminate the target antigen. T lymphocytes are essential immune system cells reportedly required for complete protection and generation of protective antibodies against virulent IBDV (57). The B cell antigenic epitopes are identified by secretory antibodies or B cell receptors to stimulate an immune response (57). Therefore, B cell epitopes are essential to induce humoral or antibody-mediated immunity, which serves as the main line of defense against severe IBDV. This approach used standard servers to identify and evaluate appropriate CTL, HTL and B cell epitopes from the targeted VP2 and VP3 proteins. Based on the evaluations, four CTL, seven HTL and 11 B cell epitopes were selected for the final vaccine construct.

The present investigation added CTB mucosal adjuvant to the final IBD-MEV construct. Adjuvants have become an essential component of most vaccines, enhancing the cell-mediated immune responses, decreasing the antigen dosage, inducing prolonged immune responses and acting as agonists for TLRs (58). The non-toxic CTB has a strong affinity for the gut mucosal GM1-ganglioside receptor (43). CTB has been used extensively in mucosal immunization strategies as a DNA vaccine adjuvant. These strategies have shown CTB as an effective adjuvant for developing mucosal antibody responses and specific immunity. Additionally, CTB activated the signaling pathways through TLR's, which are crucial in connecting innate and adaptive immunity.

To complete the final stage of the IBD-MEV construction, the epitopes and CTB adjuvant were linked using suitable flexible linkers. Linkers are crucial for improving the stability and expression of proteins in developing MEVs. The AAY linkers joining CTL epitopes enhance dissociation and epitope identification by preventing the formation of junctional epitopes. The glycine-rich GPGPG linkers that connect the HTL epitopes enhance the construct's solubility, accessibility, and flexibility of adjacent domains (30). The CTL epitopes were paired with the HTL epitopes using HEYGAEALERAG linkers, which enhance epitope presentation by creating distinct proteasomal and lysosomal cleavage sites. The bi-lysine linker that joined the B cell epitopes helps in the specific presentation of each peptide to antibodies and preserves their individual immunogenic properties (30). A rigid EAAK linker forming an alpha helix connected the CTB adjuvant to the N-terminus of the constructs to improve domain independence and stability (59). The overlapping epitope sequences were scrutinized and merged into one.

In order to confirm that the IBD-MEV construct provides an efficient immune response without eliciting allergic reactions, it is imperative to evaluate the antigenic, immunogenic and allergenic properties. The IBD-MEV was determined to be immunogenic, antigenic and non-allergenic. Generally, a promising vaccine candidate should have a molecular weight lesser than 110 kDa and an instability index lesser than 40, which classify them as relatively stable. The IBD-MEV had a molecular weight of 55.64 kDa and an instability index of 16.24, which meets the criteria for a stable vaccine. While the predicted theoretical pI of 9.24 indicates the basic nature. This may be because IBD-MEV contains basic amino acids such as arginine (4.2%), histidine (1.5%) and lysine (8.6%). The aliphatic index, which is the proportional volume occupied by the protein's aliphatic side chains, determines the thermal stability of a vaccine construct, with higher aliphatic index values indicating thermostability over a wide temperature range (60, 61). The projected aliphatic index of 86.72 for the constructed multiepitope vaccine suggested the thermostability of the protein. A key factor used to assess the protein's solubility is the Grand Average of Hydrophobicity Index (GRAVY), which represents the hydrophobicity value of a peptide. When the GRAVY value is positive, it shows hydrophobicity; when it is negative, it suggests hydrophilicity (61). The construct, with a GRAVY index of −0.256, reflects the polarity and high solubility of the IBD-MEV construct.

The IBD-MEV tertiary structure was modeled using Colabfold, a rapid protein structure and complexes prediction tool based on AlphaFold2 artificial intelligence (AI) system (46, 47). In order to predict a structure close to the native system, the 3D model needs to be refined and validated, which was achieved through the GalaxyRefine server in the study (48). The good quality of the refined model is indicated by the model's global distance test-high accuracy (GDT-HA) score, RMSD value, MolProbity score, and rotamers score. The refined model evaluated using the Ramachandran diagram showed that most of the vaccine's amino acids (96% residues) were located in the favored region. While the ProSA online server's evaluation of a *Z* score supported the vaccine's overall quality (49).

TLRs are conserved membrane-spanning proteins that function as the body's first line of defense and are essential to the innate immune system. TLRs control the transcriptional expression of cytokines by identifying pathogen-associated molecular patterns derived from pathogens (62). The cytokines production triggers the host's innate immune system to mediate antimicrobial response. Among the chicken TLRs, TLR3 tends to recognize viral dsRNA; therefore, IBD-MEV was docked against TLR3 using the HDOCK server (51, 63). The server predicted a robust interaction with a negative Gibbs-free (Δ*G*) value. The Gibbs free energy is essential to characterize the magnitude of an interaction occurring under certain circumstances in a cell. The more negative the value of the Gibbs free energy, the more energetically feasible the interaction is. Accordingly, a Δ*G* value of −295.94 kcal/mol indicates stable binding of IBD-MEV and TLR3. PDBSum revealed the existence of H-bond and salt bridge interactions between the IBD-MEV and TLR3 (64, 65). Additional validation of the docking results was performed using 50 ns molecular dynamics simulation analysis, where the root mean square deviation (RMSD) and root mean square fluctuation (RMSF) of the complex, bound and unbound IBD-MEV were determined. RMSD calculates the degree of deviation for a group of atoms to the respective initial reference structure. Thus, high RMSD values would be associated with instability in the structure. The complex structure exhibited lower RMSD trajectories as compared to MEV, indicating that IBD-MEV and TLR3 were bound in a stable and confined manner. RMSF provides more insights regarding the stability of the complex. The bound and unbound MEV structure displayed fluctuations in RMSF analysis, which may be intrinsic to the structure. These findings imply that the IBD-MEV can efficiently activate TLR3 and enhance immune defenses against the IBDV.

By modeling the host's immunological response following vaccination, immune simulations give insight into the capability of the vaccine construct against the pathogen (66). The *in silico* immune simulation results demonstrated the production of memory B cells, T cells and elevated Immunoglobulin (Ig's) levels. Upon the first IBD-MEV administration, modest production of antibodies was simulated, whereas elevated production of antibodies was observed upon the second dose. Among the immunoglobulins, high IgG and IgM levels were simulated, constituting the primary response against the virus. In addition, IgG1 + IgG2, IgG1 and IgG2 comprising the secondary and tertiary response were also noted on vaccine administration. The antigen exposure increased the B lymphocyte count, particularly the memory B lymphocytes. The progressive rise in memory B lymphocytes and immunoglobulins with repeated administration of the antigen confirms the efficacy of IBD-MEV when the host is exposed over a prolonged period of time. The T helper (TH) response exhibited a similar response, with antigen exposure increase in memory cell count was predicted. In contrast, cytotoxic T cells maintained a modest level throughout the simulation. In this way, the IBD-MEV administration simulated an efficient humoral and cell-mediated immune response against IBDV. However, further research and experimental validation of the current study's findings are necessary to confirm and validate the safety, protectivity and efficacy parameters.

## Data availability statement

Publicly available datasets were analyzed in this study. The names of the repository/repositories and accession number(s) can be found in the article/Supplementary material.

## Author contributions

IG and NS contributed to the conception and design of the study. IG and AH wrote the first draft of the manuscript. JM and TA wrote sections of the manuscript. NC supervised the *in-silico* experiments. EH, RS, NG, and SA contributed to the manuscript reading and revision. All authors approved the submitted version.
