# Supplementary material for: A multiepitope vaccine candidate against infectious bursal disease virus using immunoinformatics-based reverse vaccinology approach
Source: Front Vet Sci. 2023 Jan 13;9:1116400. doi: 10.3389/fvets.2022.1116400 (PMC9880294; doi:10.3389/fvets.2022.1116400)
Supplement: Supplementary file 1 [file Data_Sheet_1.docx]

**Supplementary:**

**Supplementary Movie:**

[TLR3-MEV Interaction Throughout The 50ns Simulation Movie](https://drive.google.com/file/d/1_Vgz_LjzGBMuomvlfPdeWAmZTB7oD0sd/view?usp=sharing)

**Supplementary Table 1:** IBDV Reference strains.

| **Reference Strain** | **Classification** | **Genogroup** | **Accession No.** |
| --- | --- | --- | --- |
| GZ29112 | Classical | 1 | AF051837.1 |
| MB11 | Classical | 1 | KU891986.1 |
| D78 | Classical | 1 | AF499929 |
| Lukert | Classical | 1 | AY918948 |
| STC | Classical | 1 | D00499.1 |
| DelE | Antigenic variant | 2 | AF133904 |
| Henan | vvIBDV | 3 | KT884486 |
| UK661 | vvIBDV | 3 | NC_004178 |
| HK46 | vvIBDV | 3 | AF092943 |
| dIBDV/UY/2014/2202 | dIBDV | 4 | KT336459 |


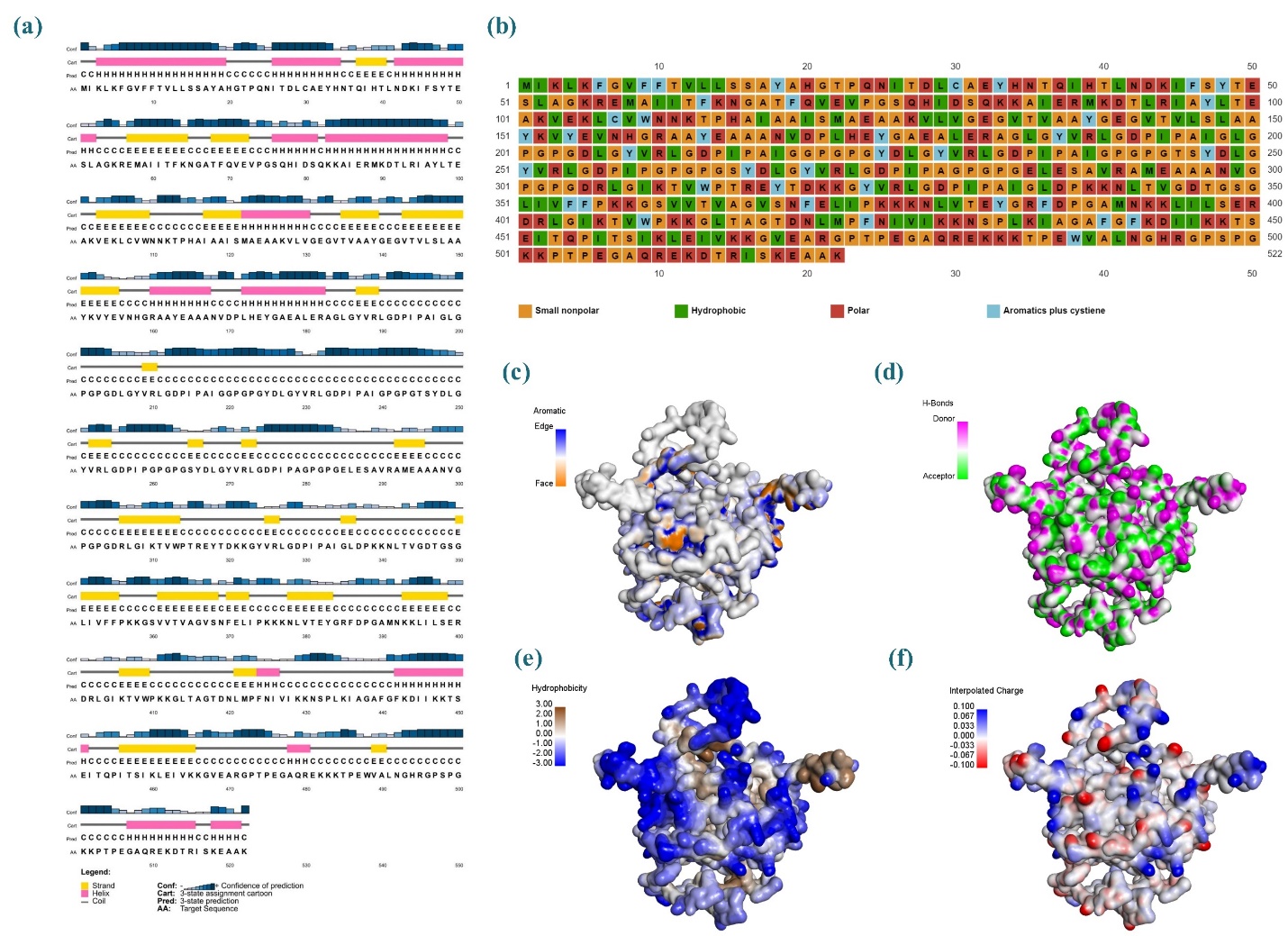


**Supplementary Figure 1:** The structural assessment of the vaccine construct **(a)** Secondary structure (strand, helix and coil) prediction; **(b)** Secondary structure residue properties; **(c)** Aromatic amino acid; **(d)** Hydrogen bonds donor-acceptor; **(e)** Hydrophobic surface and (**f**) Charge distribution.
